# The complete mitochondrial genome sequence and phylogenetic analysis of *Squalidus multimaculatus*

**DOI:** 10.1080/23802359.2020.1798297

**Published:** 2020-07-29

**Authors:** So Young Park, Sang Ki Kim, Jin Sagong, Shi-Hyun Ryu, Jeong-Nam Yu

**Affiliations:** Animal and Plant Research Department, Nakdonggang National Institute of Biological Resources, Gyeongsangbuk-do, South Korea

**Keywords:** Mitochondrial genome, phylogenetic analysis, *Squalidus multimaculatus*

## Abstract

This study reports the complete mitochondrial genome of *Squalidus multimaculatus*. The length of the mitochondrial genome of *S. multimaculatus* is 16,597 bp, including 13 protein-coding genes, 22 transfer RNA genes, two ribosomal RNA genes, and one control region. The overall base composition of A, G, T, and C is 28%, 18.8%, 25%, and 28.2%, respectively. A phylogenetic analysis by the neighbour-joining method showed a close relationship between *S. multimaculatus* and *S. japonicus coreanus*. We believe these results will provide essential data for phylogeographic studies of the genus *Squalidus*.

*Squalidus multimaculatus, S. chankaensis tsuchigae, S. japonicus coreanus,* and *S. gracilis majimae* of the *Squalidus* (Cypriniformes: Cyprinidae) genus are endemic freshwater species that are distributed in South Korea. *S*. *multimaculatus* has a restricted habitat in the rivers flowing to the East coast and is threatened with extinction (Lee et al. 2017).

In this study, we described the characterization of the complete mitochondrial genome of *S*. *multimaculatus* and analyzed its phylogenetic relationships within the genus *Squalidus*. Our study will aid in the sustainable management of this species and facilitate future research on taxonomic resolution, population genetic structure, and phylogenetic relationships.

*Squalidus multimaculatus* was collected from Gokgangcheon River, Pohang-si (N36.074631°, E129.165850°) in 2017. The voucher specimen has been archived at the Molecular Phylogenetics Laboratory in Nakdonggang National Institute of Biological Resources, Sangju-si, Korea under the accession number NNIBR-MPL2017GSM0013. The mitogenome sequence was extracted from the next-generation sequencing data (unpublished data) using the Illumina HiSeq 4000 platform by GnC Bio (Daejeon, Korea). In total, 158 Gb raw reads were obtained and assembled using the Platanus assembler (ver.1.2.4., Kajitani et al. 2014). In total, 77,028 reads were used to reconstruct the mitogenome using Deconseq (ver.0.4.3., Schmieder and Edwards 2011) and it was annotated using gsMapper (ver. 2.8.; Roche) using MitoFish database as the reference (Sato et al. 2018).

The complete mitogenome of *S*. *multimaculatus* is 16 597 bp (GenBank accession number: MK840865) in length, consisting of 13 protein-coding genes (PCGs), 22 transfer RNA (tRNA) genes, two ribosomal RNA (rRNA) genes, and one control region (D-loop). The control region was 924 bp in length and located between the tRNA^Pro^ and tRNA^Phe^. The overall base composition was 28% of A, 18.8% of G, 25% of T, 28.2% of C, and had a slight AT bias of 53%. All the PCGs used the start codon ATG except *COI*, which used GTG; this is in accordance with the mitochondrial genome analysis of 250 fishes performed by Satoh et al. Five genes (*COI*, *ATPase8*, *ND4L*, *ND5*, and *ND6*) used TAA as a stop codon, *ND1* used TAG as a stop codon, whereas the remaining seven genes used incomplete stop codons (T–– or TA–). It is speculated that having 3′ end PCGs followed by the tRNA gene encoded on the same strand may permit transcription to terminate without complete stop codons (Satoh et al. 2016). The 12S and 16S rRNAs were 959 bp and 1685 bp, respectively. The tRNA sequence length ranged from 69 to 76 bp; all the tRNA genes, which were predicted using MITOS (Bernt et al. 2013), had a three-leaf clover structure, except tRNA-Ser^UCG^.

To examine the phylogenetic position of *S*. *multimaculatus*, we downloaded the mitochondrial genome sequences of five species belonging to the genus *Squalidus* from GenBank (Tang et al. 2011; Li et al. 2016; Liu et al. 2016; Zhou et al. 2016; Park et al. 2016, 2020; Yi et al. 2017). The phylogenetic tree was constructed with the sequences of the 13 PCGs using the neighbor joining method in the program MEGA7. A close relationship was observed between *S*. *japonicas coreanus* and *S*. *multimaculatus* (KX495606.2, and in this study; Figure 1). *Squalidus multimaculatus* has a restricted habitat, hence, more genetic and phylogeographic studies are needed to preserve this species. Our study provides useful information that may contribute toward future phylogeographic studies in the *Squalidus* species.

**Figure 1.**
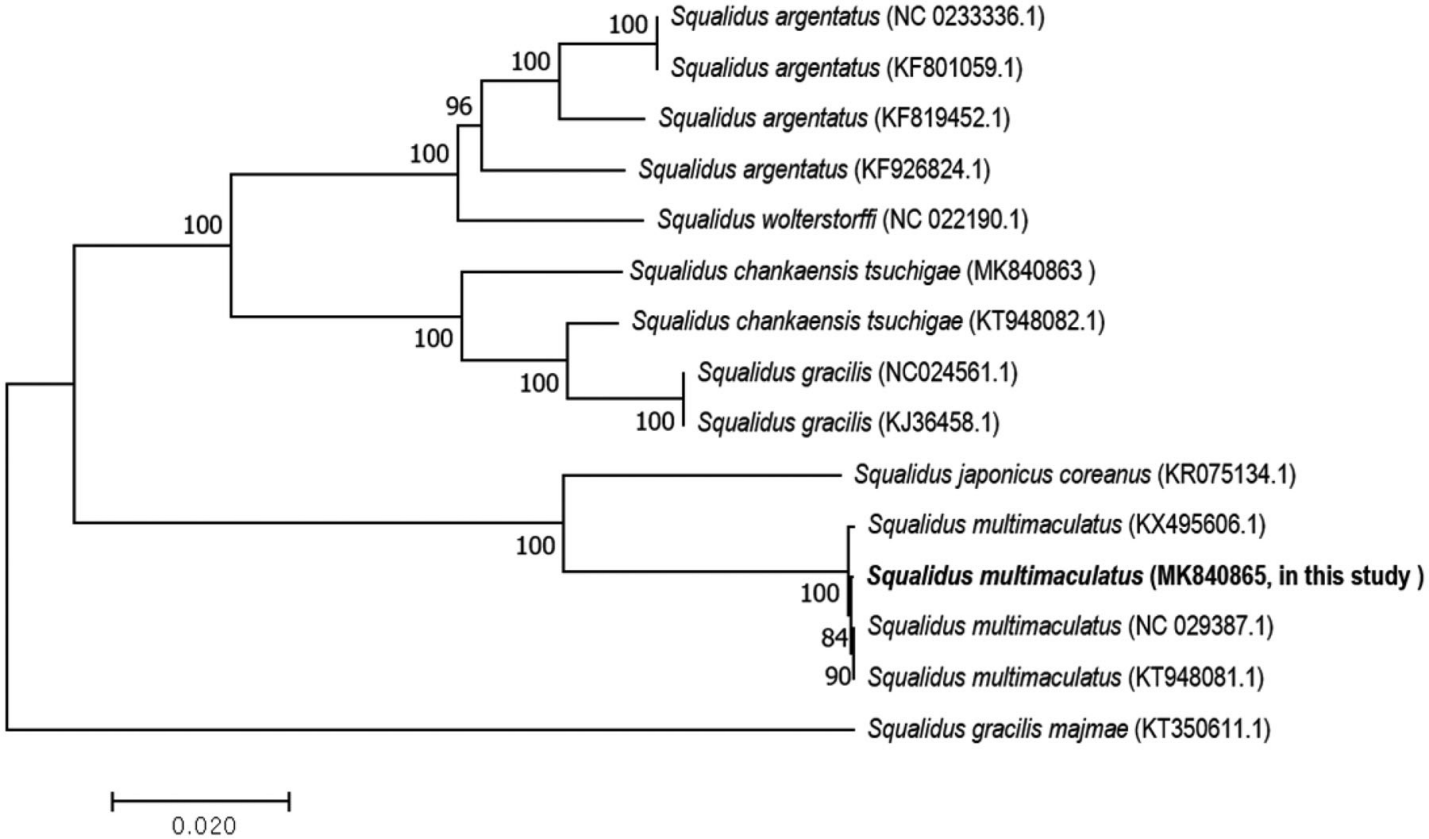
A phylogenetic tree construction of five species based on 13 protein-coding gene sequences of complete mitochondrial genome sequences accessed from GenBank using the neighbor-joining method. The tree was computed using Kimura’s 2-parameter distance model with bootstrap value: 10,000. The scale bar indicates 0.02 substitutions per nucleotide position.

## Data Availability

The data that support the findings of this study are available in GenBank, NCBI at https://www.ncbi.nlm.nih.gov under the accession number: MK840865.
